# Metropolitan Hospital Sunday Fund

**Published:** 1908-12-26

**Authors:** 


### METROPOLITAN HOSPITAL SUNDAY FUND.

### ANNUAL MEETING OF CONSTITUENTS.

Another Anti-Vivisectionist Protest.

The annual meeting of the constituents of the Metropolitan Hospital Sunday Fund was held at the Mansion House on Wednesday, December 16.

The Lord Mayor, Sir George Wyatt Truscott, presided, and, in opening the proceedings, said he had pleasure in welcoming the constituents of the Fund to the Mansion House, and in congratulating them on having, during the past year, had the largest collection towards the Fund. The collection reached a total exceeding £80,000, and this in spite of the fact that the collection from places of public worship had fallen off by over £2,500 as compared with last year. This falling off might, no doubt, be accounted for very largely by the holding of the Pan-Anglican Congress, for, although it was intended that no existing fund should suffer as a consequence of the Congress, he was afraid that they had. They hoped however, that this was only a passing phase in the history of the Fund and that his successor might be able to congratulate himself on a record collection from all sources.

Death of Canon Fleming and Dr. Glover.

They would remember that last year the resolution to hold Hospital Sunday on June 21 was moved by Canon Fleming and seconded by Dr. Glover. He knew that they would join him in an expression of the deepest regret that since that time both those able supporters of the Fund had passed away. No man ever pleaded the cause of Hospital Sunday with more eloquence or to better purpose than the late Canon Fleming. He bore the name of "the silver tongued Fleming," and he employed that silver tongue in the cause of charity with immense effect. During the thirty-six years of his ministry the collections taken from his congregations amounted to £35,467, or an average of nearly £1,000 a year. In Dr. Glover they had lost one of the founders, and they could not but regard his loss with the deepest regret.

Sir John Bell (ex Lord Mayor), in moving the adoption of the report of the Council, said he desired very briefly to endorse what the Lord Mayor had said regarding the late Canon Fleming and Dr. Glover, whose memories would always be cherished by those who remained to fill their places.

The Church Collections.

Although the Fund had been prosperous during the year it had only been so as a consequence of large donations from the Herring Trust and other funds. If it had not been for this he was afraid they would not have performed their part so well. He had no doubt the clergy would continue their work and even extend their sphere of usefulness. (Cheers.)

The Earl of Stamford, in seconding, said that although the Fund had fallen off as a consequence of the holding of the Pan-Anglican Congress, yet he had it on very high authority that there had been counter-balancing elements, and if their friends in the Church of England had solved any of the difficult problems in connection with the Church the falling off in the collection was not to be altogether deplored. (Cheers.)

The Anti-Vivisectionists.

The Rev. L. S. Lewis (Vicar of St. Mark's, Whitechapel) said he rose to point out a blot on the report.

The Lord Mayor: Do you intend to move an amendment?

Mr. Lewis: I rose, as I said, to point out a blot on the report and to move a protest.

The Lord Mayor: If you intend to move an amendment you must submit it now, because I think you are out of order.

Mr. Lewis: I move that the report of the Distribution Committee be sent back for reconsideration.

The Lord Mayor: For what purpose?

Mr. Lewis: I always understood that no argument should be embodied in an amendment, and you must permit me to develop my argument in what I have to say.

The Lord Mayor: I do not think this can be in order.

Mr. Lewis: On page eighty-nine of this report it is shown that no award is recommended for the National Anti-Vivisection Hospital at Battersea.

The Lord Mayor: I do not think this question can be discussed now. It is a matter for the Committee of Distribution.

Mr. Lewis: With great respect, sir, may I direct your attention to the rule in which it is stated that all congregations which have forwarded contributions to the Fund during two successive years shall be entitled to a voice in the management during the year following the last of such contributions, and the minister and two laymen shall be summoned as forming constituents of the Fund to meet the Council. We held a vestry meeting to consider this point, and a lay member and myself were sent as a deputation to bring this blot on the management of the Fund before your notice.

The Lord Mayor: I consider this is out of order.

Mr. Lewis: Will you allow me to say that this is contrary to the spirit in which this Fund should be managed?

The Lord Mayor: This is a matter which ought to be put before the Committee, and not before this meeting.

Mr. Lewis: Then we have no voice in the management of this Fund?

The Lord Mayor: This matter was raised last year, and was ruled out of order.

Mr. Lewis: I was very badly treated last year, but the matter was discussed, and was not ruled out of order. Your own minutes show this.

Rev. A. Grundy: This gentleman is such an enthusiast that he and his congregation send the Fund 3s. 3d. (Cheers.)

Mr. Lewis: It is a poor parish.

Sir James Bell: I appeal to the Lord Mayor to say whether a contribution of 3s. 3d. entitles this gentleman to raise this question. (Mr. Lewis: Shame.) I agree with you, sir, it is a shame that you should come here for no other purpose than that of interfering with this meeting. (Cheers.) You are not here to use your influence in the interest of hospitals or of charity. (Cheers.)

Mr. Lewis: To what resolution is this gentleman speaking?

Sir James Bell: I have no desire to bandy words with this gentleman, but to appeal to the Lord Mayor and the meeting whether a collection of 3s. 3d. entitles this gentleman and his congregation to interfere with the meeting?

The Lord Mayor: I rule this out of order.

The resolution for the adoption of the report was then put to the meeting, and was carried, with two dissentients.

### Hospital Sunday, 1909.

The Rev. C. H. Grundy moved that Hospital Sunday be held on June 13 next, and that the clergymen of all denominations be asked to co-operate with the Council in the usual way. They had been told that the falling-off in the collection was due to the holding of the Pan-Anglican Congress. He could have wished that the Congress should have considered the home Church, as well as the Church abroad. There would have been more of the "Pan" about it if it had. (Laughter.) He would have liked to see the Congress give one-third of its collections to such purposes as theirs, but as it was it panned out so that the foreign Church took it all. The fact that the collections in churches fell off by £2,500 was, in his opinion, an absolute disgrace to the clergy of all denominations. He thought they had not been doing their duty. But while he blamed the clergy, he also blamed the churchwardens, and he hoped it would not occur again. (Laughter and cheers.)

The Rev. Hardy Harwood said he did not regard the falling-off in the revenue from collections as such a calamity as previous speakers had represented. The fact that, in spite of the Congress and many other drains upon the public during the year, there had been a decrease of only £2,500 showed that the Fund had an exceedingly steady source of revenue. It should be remembered that, with the exception of some large contributions from trusts, the bulk of the collection came from the poorer classes. To his mind Hospital Sunday was one of the holiest and most helpful days of the whole year. (Cheers.) He desired, however, to make a suggestion. The Fund appealed only to worshipping congregations, but since the early days of the Fund a large number of Sunday meetings were now held in concert halls and other places, and he thought the Council might also appeal to them.

The resolution was adopted, as was also a vote of thanks to the Chairman, proposed by Archdeacon Sinclair and seconded by Sir William Church.

The Lord Mayor, in returning thanks, said that when his father occupied the chair at the same meeting in 1879 the total revenue of the Fund was only £26,500. Now it was over £80,000, so that in twenty-nine years it had more than trebled. (Cheers.)

